# Silencing of TLR4 Inhibits Atrial Fibrosis and Susceptibility to Atrial Fibrillation via Downregulation of NLRP3-TGF-*β* in Spontaneously Hypertensive Rats

**DOI:** 10.1155/2022/2466150

**Published:** 2022-07-11

**Authors:** Chenliang Ge, Yaxin Zhao, Yuming Liang, Yan He

**Affiliations:** ^1^Department of Geriatrics Cardiology, The First Affiliated Hospital of Guangxi Medical University, Nanning 530021, China; ^2^Internal Medicine-Cardiovascular Department, Jiangbin Hospital, Nanning 530021, China

## Abstract

**Introduction:**

This study was aimed at exploring whether silencing of TLR4 could inhibit atrial fibrosis and susceptibility to atrial fibrillation (AF) by regulating NLRP3-TGF-*β* in hypertensive rats.

**Methods:**

Spontaneously hypertensive rats (SHRs) were transfected with either a virus containing TLR4-shRNA to downregulate TLR4 or an empty virus (vehicle) at the age of 14 weeks. Fibrosis of left atrium and susceptibility to AF were detected, and expression of NLRP3-TGF-*β* in left atrial tissue at 22 weeks of age was measured. Primary cardiac fibroblasts were transfected with TLR4-shRNA or scrambled vehicle and stimulated with angiotensin (Ang) II. Proliferation of cardiac fibroblasts and expression of NLRP3-TGF-*β* were detected.

**Results:**

Silencing of TLR4 reduced left atrial fibrosis and susceptibility to AF in SHRs and downregulated expression of NLRP3, TGF-*β*, and collagen I. In vitro, TLR4 silencing reduced proliferation of cardiac fibroblasts induced by Ang II as well as expression of NLRP3, TGF-*β*, and collagen I.

**Conclusion:**

Silencing of TLR4 can downregulate NLRP3-TGF-*β* to reduce atrial fibrosis and susceptibility to AF in SHRs.

## 1. Introduction

Hypertension is an important risk factor for cardiovascular events as it creates a greater pressure load on the heart and may cause left atrial fibrosis and dilation that can lead to heart failure and atrial fibrillation (AF) [1, 2]. AF and hypertension are significantly associated with a higher risk of cardiovascular disease. Hypertension is common in humans and constitutes the great risk of developing AF. The prevalence of hypertension is approximately 60–80% among patients with established AF [3, 4]. Currently, the specific pathophysiological mechanism by which hypertension causes atrial fibrosis and AF remains elusive.

Toll-like receptor 4 (TLR4) has been shown to be upregulated under hypertension [5–8]. TLR4 and nucleotide-binding domain leucine-rich repeat family pyrin domain-containing 3 (NLRP3) protein are vital regulators of the innate immune system. Increased expression of TLR4 is related to development and maintenance of hypertension [9]. Recent research revealed increased TLR4 expression in spontaneously hypertensive rats (SHRs) compared to Wistar rats, while reduced hypertension and myocardial fibrosis were observed when TLR4 activity was inhibited [10–13]. In response to cardiovascular injury, the NLRP3 inflammasome acts downstream of TLR4 to activate interleukin- (IL-) 1*β* and IL-18 [14]. Previous studies identified NLRP3 inflammasome as being involved in the pathophysiological process of ventricular remodeling, which was improved upon inhibition of NLRP3 [15, 16]. In addition, activation of NLRP3 inflammasome and secretion of IL-1*β* and IL-18 lead to atrial fibrosis and increased susceptibility to AF [17–19]. Transforming growth factor *β* (TGF-*β*) is a recognized therapeutic target for treatment of organ fibrosis. TGF-*β* can facilitate synthesis of collagens and fibronectins, which induces protease inhibitors and leads to extracellular matrix deposition [20, 21]. Thus, we speculated that silencing of TLR4 might inhibit the NLRP3-TGF-*β* signaling pathway to alleviate atrial fibrosis and reduce susceptibility to AF.

Hypertension-associated atrial remodeling has been linked to increased susceptibility to AF in SHRs [5]. The aim of the study was to explore whether silencing TLR4 can downregulate expression of NLRP3-TGF-*β* to inhibit atrial fibrosis and reduce susceptibility to AF in SHRs. The effect of silencing TLR4 on proliferation of cardiac fibroblasts in vitro was also investigated.

## 2. Materials and Methods

### 2.1. Experimental Animals and Experimental Groups

Animal care and all experimental procedures were approved by the Ethics Committee of Guangxi Medical University. All rats were ordered from Charles River Laboratories (Beijing, China) and weighed about 310–340 g. The rats were fed with ordinary chow, housed at 22 ± 2°C, maintained on a 12 h light/dark cycle, and adaptively reared for 1 week. Eighteen 14-week-old SPF male SHRs were assigned into SHR, SHR+vehicle, and SHR+TLR4-shRNA groups, and the same age homologous Wistar rats were assigned to the control group (*n* = 6 for each).

TLR4 was silenced in SHRs by lentiviral shRNA transfection. The lentivirus-mediated TLR4-shRNA and a lentivirus carrying scrambled shRNA (lenti-vehicle) as a negative control were synthesized by Genechem (Shanghai, China). Sequences for rat TLR4-shRNA oligos were forward: aaCCTAGAACATGTGGATCTT and reverse: AAGATCCACATGTTCTAGGTT. In the SHR+vehicle and SHR+TLR4-shRNA groups, vehicle (empty virus) and TLR4-shRNA (titer: 1 × 10^9^ TU (transduction units)/mL) were administered by jugular intravenous injection at 14 weeks of age. All rats were fed up to 22 weeks of age. Rats were weighed by an electronic animal scale and tested for systolic blood pressure and heart rate by monitoring caudal arteriopalmus using a BP-2010A intelligent noninvasive blood pressure monitor (Softron Biotechnology Co., Ltd., Beijing, China) before and after the experiments. All measurements were repeated 3 times.

### 2.2. AF Susceptibility

Isoflurane (5% induction and 2–3% maintenance) was administered to rats to induce anesthesia, and body temperature was maintained at 37°C with a heated pad. Rats were connected a standard lead II electrocardiogram using the Medlab electrocardiogram (ECG) signal acquisition and processing system (Nihon Kohden Corp, Tokyo, Japan). Transesophageal burst rapid pacing was performed to evaluate susceptibility to AF. A clinically available 6F 10 pole coronary sinus electrode catheter (ten 0.5 mm circular electrodes, interelectrode distance 2.0 mm, and electrode pair spacing 6.0 mm) was inserted into the esophagus and positioned at a location where the lowest threshold could capture the atrium. To induce AF, a series of 5 consecutive bursts of rapid stimulation (25, 30, 40, 50, and 83 Hz) was applied for 30 seconds, with each series being separated by 5 minutes. AF was defined as an abnormal ECG with an absolute irregular RR interval and a fragmented and rapid P wave for at least 2 seconds immediately following burst pacing. P wave maximum duration (PMD) was obtained by measuring the maximum distance between the beginning and end of the P wave in the limb leads. The number and duration of inducible AF episodes were recorded. AF susceptibility was calculated as the ratio of the number of AF episodes to the total number of procedures. AF duration was the time from the end of burst pacing to the first sinus P wave following atrial rhythm. We defined the total time of an AF episode as the sum of the AF durations of all the episodes [22, 23].

### 2.3. Hematoxylin and Eosin (HE) and Masson's Trichrome Staining

Upon induction of anesthesia, the heart was isolated from the chest cavity and flushed repeatedly with 0.9% sodium chloride. The left atrium was separated and fixed in 4% paraformaldehyde overnight. The sample tissue was extracted and washed the following day. Dehydration was carried out by 75% ethanol for 1 h followed by 85% ethanol for 15 s, two 95% ethanol rinses over 45 min, three 100% ethanol rinses over 40 min, and xylene for 3–5 min. Then, the tissue samples were waxed, sliced, and stained with HE and Masson's trichrome. The collagen volume fraction (CVF) of each visual field in three randomly selected nonoverlapping fields of Masson-stained sections was calculated using ImageJ software. CVF was calculated as the ratio of collagen area to the total visual field, and an average value was calculated from the three visual fields.

### 2.4. Immunohistochemical (IHC) Staining

The samples were blocked with goat serum for 30 min, after which rabbit anti-type I collagen polyclonal antibody (Proteintech, 14695-1-AP, 1 : 200, Wuhan, China) and rabbit anti-type III collagen polyclonal antibody (Proteintech, 22734-1-AP, 1 : 200, Wuhan, China) were added and incubated overnight in a humid box at 4°C. On the following day, the samples were placed at room temperature for rewarming, washed, and incubated with biotin-labeled goat anti-rabbit IgG at room temperature. The tissue slices were then incubated with horseradish peroxidase- (HRP-) labeled streptomycin albumin working solution followed by DAB color development solution. Brown particles visible by light microscopy indicated a positive result. Three visual fields of each section were selected at random for photography and analyzed by ImageJ software. The area ratio of collagen expression was calculated as the ratio of the area occupied by type I collagen or type III collagen to the total area of the visual field.

### 2.5. Extraction and Culture of Primary Cardiac Fibroblasts (CFs)

12 Sprague-Dawley (SD) neonatal rats (1–3 days old) were purchased from Guangxi Medical University Laboratory Animal Center. The heart was removed and the left atrium was placed in a culture dish on ice, washed to remove blood and redundant tissues, and then transferred to culture medium supplemented with 10% fetal bovine serum. The tissue was cut into pieces (1 mm^3^) with the scissors, washed with phosphate buffered saline (PBS), and treated with 0.08% type II collagenase and 0.25% trypsin. After reaction for 5 min in an incubator at 37°C, 0.5 ml culture medium was added to halt digestion, and the supernatant was sampled after 1–2 min and centrifuged at 1000 rpm for 10 min. After centrifugation, the supernatant was discarded, and the remaining cells were cultured in fresh culture medium in an incubator. After 90 min of culture using the differential adhesion method, the medium was discarded and the floating cells were removed. The cells were passaged until the density reached 85–90%.

### 2.6. Identification of CFs by Immunofluorescence Staining

Vimentin, a biological marker of CFs, was detected by immunofluorescence [24, 25]. Cells inoculated in a 6-well plate were fixed with 4% paraformaldehyde at room temperature for 15 min and washed with PBS 3 times for 5 min each. The cells were then processed by the following reagents: 0.25% Triton permeabilization reagent (diluted with pure water) at 37°C for 10 min, PBS 3 times for 5 min each, 10% goat serum blocking solution at 37°C for 30 min diluted with PBS, primary vimentin antibody (Proteintech, 10366-1-AP, 1 : 200, Wuhan, China) at 4°C overnight, PBS 3 times for 10 min each, fluorescence-labeled goat anti-rabbit secondary antibody (Cell Signaling Technology, 4412, 1 : 200) at 37°C for 45 min, PBS 3 times for 8 min each, DAPI solution for 5 min, and PBS 3 times for 3 min each. Results were observed and photographed under a confocal fluorescence microscope.

### 2.7. Proliferation of CFs Detected by CCK-8 (Cell Counting Kit-8)

To determine the effect of Ang II on proliferation of cardiac fibroblasts, cell proliferation was detected using CCK-8 to measure the number of viable cells. Digested cells were counted and suspended at 5 × 10^4^ cells/mL before seeding 100 *μ*L/well into a 96-well plate. After 24 h, the cells were starved for another 24 h, followed by addition of Ang II (0, 10^−5^, 10^−6^, 10^−7^, 10^−8^, or 10^−9^ mol/L). Within 24 h, CCK-8 solution (10 *μ*L/well) was added and the cells were then incubated for 2 h. A microplate reader was used to measure absorbance of each well at 450 nm.

### 2.8. Experimental Groups

Cells were seeded in six-well plates at a density of 5 × 10^4^/mL. The culture medium used for primary cardiac fibroblasts was replaced by serum-free DMEM (Dulbecco's modified Eagle's medium) to starve them for 12 h. Cells were divided into 4 groups: control, Ang II, Ang II+vehicle, and Ang II+TLR4-shRNA. Ang II+vehicle and Ang II+TLR4-shRNA were transfected with the shRNA negative control (vehicle) and TLR4-shRNA (10^8^ TU/mL and multiplicity of infection = 50). Then, cells in the Ang II, Ang II+vehicle, Ang II+TLR4-shRNA groups were starved for 24 h before being stimulated by 10^−6^ mol/L Ang II for 24 h.

### 2.9. Quantitative Real-Time PCR

Total RNA from left atrial tissue and fibroblasts was collected according to the manufacturer's instructions for the RNA Extraction Kit (TaKaRa, 9767, Beijing, China). Total RNA (1 *μ*g) from each sample was reverse-transcribed into cDNA using PrimeScript RT Master Mix (TaKaRa, RR036A, Beijing, China) for quantitative real-time PCR, which was performed in a 7500 real-time fluorescence quantitative PCR system (Thermo Fisher Scientific, USA) using TB Green Premix (TaKaRa, RR820A, Beijing, China). PCR was carried out under the following conditions: 95°C for 30 s followed by 45 cycles at 95°C for 10 s and 60°C for 30 s. Data were expressed as a ratio of the signal from the band of interest to that of the GAPDH band; the latter acted as the internal control in the experiment. Relative mRNA expression levels were described as the 2-*ΔΔ*Ct value. The sequences of the primers used for PCR are listed in Table 1.

### 2.10. Western Blot

Total protein was extracted from left atrial tissue and atrium fibroblasts using RIPA Lysis Buffer (Beyotime, P0013B, Beijing, China). Protein concentration was measured with the BCA Protein Assay kit (Beyotime, P0010, Beijing, China). Protein (25 *μ*g) was separated via 10% SDS-PAGE at 70 V for 1 h and then transferred onto PVDF membranes at 220 mA for 2 h. The membranes were blocked with 5% nonfat powdered milk in tris-buffered saline with tween (TBST) for 1 h at room temperature. The blot was incubated overnight at 4°C with primary antibodies targeting TLR4 (Abcam, ab22048, diluted 1 : 1000), NLRP3 (NOVUS, NBP2-12446, diluted 1 : 250), pro-caspase-1 (Abcam, ab179515, diluted 1 : 1000), caspase-1-p20 (Proteintech, 22915-1-AP, diluted 1 : 1000), TGF-*β* (Cell Signaling Technology, 3711, diluted 1 : 1000), IL-1*β* (Cell Signaling Technology, 31202, diluted 1 : 1000), IL-18 (Wanleibio, diluted 1 : 1000, Shenyang, China), collagen I (Proteintech, 14695-1-AP, diluted 1 : 2000), or GAPDH (Cell Signaling Technology, 5174, 1 : 2000). The membranes were then washed with TBST three times and incubated with the corresponding secondary antibodies for 1 h at room temperature. GAPDH was used as an internal control. The western blot bands were imaged by an enzymatic chemiluminescence (ECL) kit (Solarbio, PE0010, Beijing, China) and quantified using ImageJ software.

### 2.11. Enzyme-Linked Immunosorbent Assay (ELISA)

Concentrations of IL-1*β* and IL-18 in cell culture supernatants were determined by enzyme-linked immunosorbent assay (ELISA). Following the manufacturer's instructions for rat IL-1*β* (Sangon Biotech, D731007, Shanghai, China) and IL-18 (Sangon Biotech, D731079) ELISA kits, standards and samples were added to a microplate that had been precoated with anti-rat IL-1*β* and IL-18 antibodies. After incubation, biotinylated anti-rat IL-1*β* and IL-18 antibody were added to the microplate and combined with HRP-conjugated streptavidin to form an immune complex and then washed five times to remove unbound enzyme. Chromogenic substrate was added to produce a blue color and then converted to the final yellow color by acid. Finally, absorbance (OD) was measured at 450 nm. The concentration of IL-1*β* and IL-18 in the sample was proportional to the OD.

### 2.12. Statistical Analysis

All data were expressed as mean ± SEM and statistical analysis was carried out with SPSS 22.0 software. Data of each group were tested for normal distribution and homogeneity of variance before further analysis. Independent sample *t* tests or one-way analysis of variance (ANOVA) was performed for comparisons between two or more groups of continuous variables. A post hoc Tukey's test was used for pairwise comparison between groups, while the chi-square test was used when comparing rates. *P* ≤ 0.05 indicated a statistically significant difference.

## 3. Results

### 3.1. Body Weight, Heart Rate, and Blood Pressure of Rats

**Figure 1:**
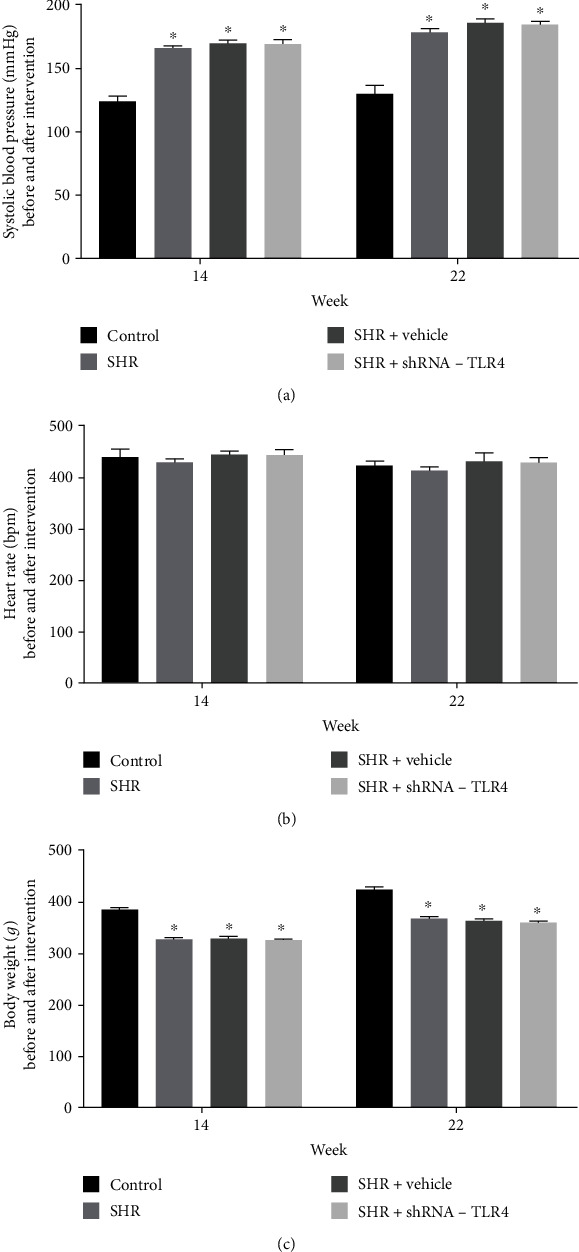
Systolic blood pressure, heart rate, and body weight of rats. Systolic blood pressure (a), heart rate (b), and body weight (c) before (14 weeks) and after (22 weeks) intervention. *n* = 6 in each group. Data are expressed as the mean ± SEM. Statistical analyses: *P* values were evaluated by ANOVA, followed by a post hoc Tukey's test. ^∗^Compared with control; *P* < 0.001 indicates a significant difference.

Systolic blood pressure in the SHR, SHR+vehicle, and SHR+TLR4-shRNA groups was significantly higher than in the control group, although there were no remarkable changes in blood pressure in any of these groups between 14 and 22 weeks old (Figure 1(a)). There were no significant differences in heart rate between the four groups, while body weight in the control group was significantly greater than in the SHR, SHR+vehicle, and SHR+TLR4-shRNA groups at 14 and 22 weeks of age (Figures 1(b) and 1(c)).

### 3.2. Silencing of TLR4 Reduces Susceptibility to AF

**Figure 2:**
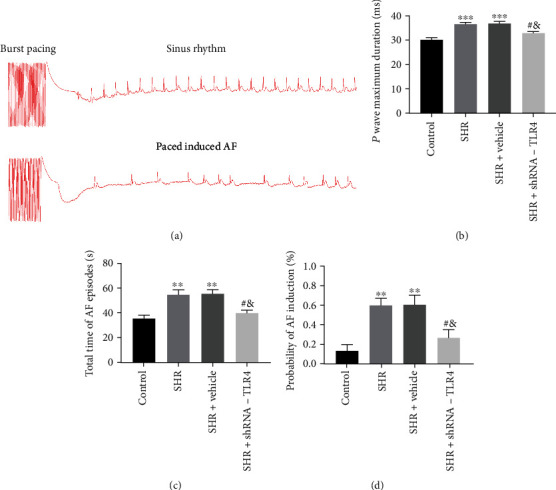
Silencing of TLR4 alleviated susceptibility to atrial fibrillation (AF) in spontaneously hypertensive rats. (a) Typical surface electrogram lead II recordings of sinus rhythm and induced atrial fibrillation after burst pacing. (b–d) Statistically significant results of P wave maximum duration, total time of atrial fibrillation episodes, and probability of induced AF in each group (*n* = 6 for each). Data are expressed as mean ± SEM. Statistical analyses: *P* values were evaluated by ANOVA, followed by a post hoc Tukey's test. ^∗^*P* < 0.05, ^∗∗^*P* < 0.01, and ^∗∗∗^*P* < 0.001 versus the control; ^#^*P* < 0.05, ^##^*P* < 0.01, and ^###^*P* < 0.001 versus the SHR group; ^&^*P* < 0.05 versus the SHR+vehicle group; significantly different as indicated.

Before transesophageal burst rapid pacing, no differences were observed in ECGs between the 4 groups of rats and no AF was observed at baseline. Representative electrocardiograms are shown for the AF susceptibility protocol in Figure 2(a). P wave maximum duration, total time of AF episodes, and probability of induced AF are depicted in Figures 2(b)–2(d). The SHR group exhibited a longer maximum P wave duration, a longer total time of AF episodes, and a significantly increased probability of induced AF compared with the control group. P wave maximum duration, total time of AF episodes and probability of induced AF were reduced in the SHR+TLR4-shRNA group compared with the SHR group, while no differences were observed between the SHR and SHR+vehicle groups (Figures 2(b)–2(d)).

### 3.3. Silencing of TLR4 Alleviates Atrial Fibrosis

**Figure 3:**
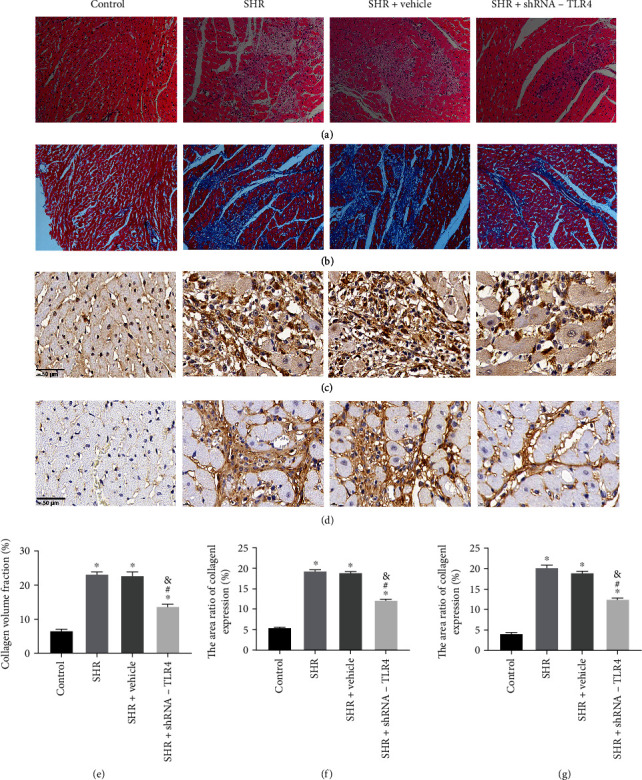
Silencing of TLR4 alleviated atrial fibrosis. (a) Representative hematoxylin-eosin (HE) stain of rat left atrial tissue sections (200x). (b) Representative Masson's trichrome stain of rat left atrial tissue sections (200x). (c) Expression of collagen I protein was detected by immunohistochemical (IHC) staining in left atrial tissue; scale bar = 50 *μ*m. (d) IHC staining for collagen III in magnified views; scale bar = 50 *μ*m. (e) Volume fraction of collagen was quantified by Masson stain; data are representative of six samples per group. (f) Expression of collagen I protein was quantified by immunohistochemistry; data are representative of six samples per group. (g) Expression of collagen III protein was quantified by immunohistochemistry; data are representative of six samples per group. All the aforementioned data are represented as mean ± SEM. Statistical analyses: *P* values were evaluated by ANOVA, followed by a post hoc Tukey's test. ^∗^*P* < 0.001 versus the control group; ^#^*P* < 0.001 versus the SHR group; ^&^*P* < 0.001 versus the SHR+vehicle group; significantly different as indicated.

HE staining showed inflammatory cell infiltration in the SHR and SHR+vehicle groups. Compared with those two groups, there were fewer pathological changes in the SHR+TLR4-shRNA group (Figure 3(a)). Masson's trichrome staining revealed that left atrial fibrosis in the SHR and SHR+vehicle groups was significantly aggravated relative to that in the control group. There were no remarkable differences between the SHR group and SHR+vehicle groups, while a significant reduction was observed in the SHR+TLR4-shRNA group compared with the SHR and SHR+vehicle groups (Figures 3(b) and 3(e)). IHC results showed significantly increased collagen I and collagen III expressions in the SHR and SHR+vehicle groups compared with the control group. No differences were observed between the SHR and SHR+vehicle groups, but expression of collagen I and collagen III was reduced in the SHR+TLR4-shRNA group compared with the SHR and SHR+vehicle groups (Figures 3(c), 3(d), 3(f), and 3(g)).

### 3.4. Silencing of TLR4 Inhibited Activation of NLRP3 Inflammasome and Expression of TGF-*β* In Vivo

**Figure 4:**
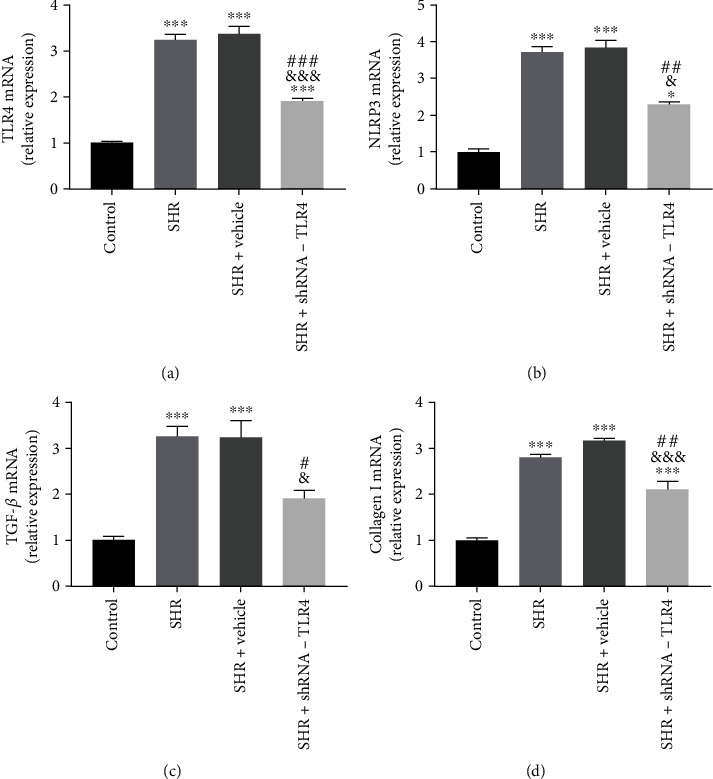
Silencing of TLR4 reduced mRNA expression of NLRP3 and TGF-*β* in vivo. Relative mRNA expression of TLR4, NLRP3, TGF-*β*, and collagen I normalized to GAPDH in left atrial tissue (a–d). All aforementioned data are represented as mean ± SEM (*n* = 3). Statistical analyses: the *P* values were evaluated by ANOVA, followed by a post hoc Tukey's test. ^∗^*P* < 0.05, ^∗∗^*P* < 0.01, and ^∗∗∗^*P* < 0.001 versus the control; ^#^*P* < 0.05, ^##^*P* < 0.01, and ^###^*P* < 0.001 versus the SHR group; ^&^*P* < 0.05, ^&&^*P* < 0.01, and ^&&&^*P* < 0.001 versus the SHR+vehicle group; significantly different as indicated.

Expression of TLR4 mRNA in the SHR+TLR4-shRNA group was significantly lower than the SHR and SHR+vehicle groups, indicating successful lentiviral transfection and knockdown of the TLR4 gene (Figure 4(a)). A significant increase in TLR4, NLRP3, TGF-*β*, and collagen I mRNA expressions was observed in the SHR and SHR+vehicle groups compared with the control group. There were no differences in expression between the SHR and SHR+vehicle groups, and expression of TLR4, NLRP3, TGF-*β*, and collagen I mRNA in the SHR+TLR4-shRNA group was decreased compared with the SHR group (Figures 4(b)–4(d)).

**Figure 5:**
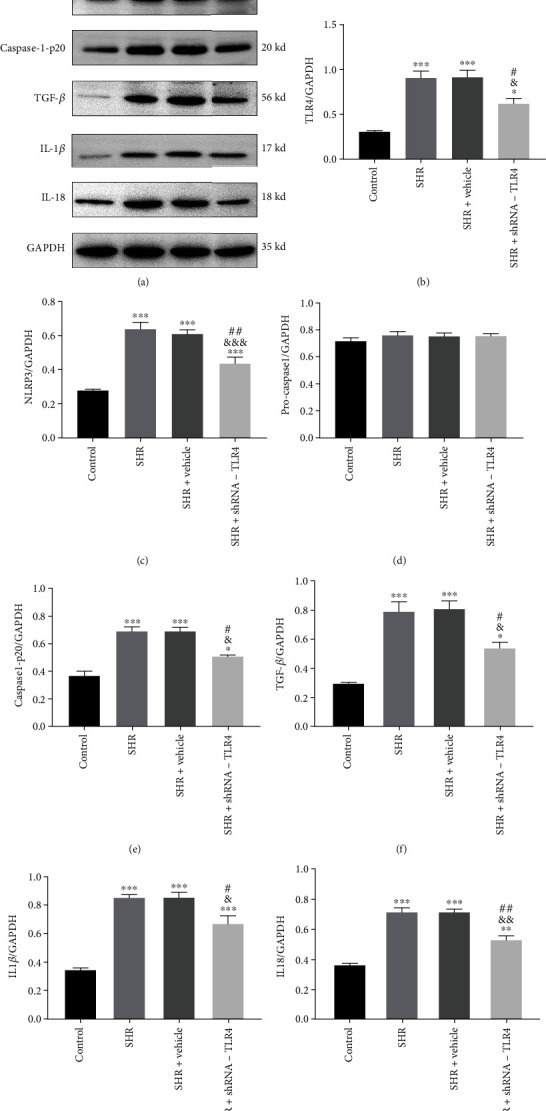
Silencing of TLR4 reduced expression of NLRP3 and TGF-*β* proteins in vivo. Protein expression of TLR4, NLRP3, pro-caspase-1, caspase-1-p20, TGF-*β*, IL-1*β*, and IL-18 detected by western blots in left atrial tissue. (a) Representative western blots show expression of TLR4, NLRP3, pro-caspase-1, caspase-1-p20, TGF-*β*, IL-1*β*, and IL-18. (b–h) Relative protein expression of TLR4, NLRP3, pro-caspase-1, caspase-1-p20, TGF-*β*, IL-1*β*, and IL-18 normalized to GAPDH in left atrial tissue. All the aforementioned data are represented as mean ± SEM (*n* = 3). Statistical analyses: *P* values were evaluated by ANOVA, followed by a post hoc Tukey's test. ^∗^*P* < 0.05, ^∗∗^*P* < 0.01, and ^∗∗∗^*P* < 0.001 versus the control; ^#^*P* < 0.05, ^##^*P* < 0.01, and ^###^*P* < 0.001 versus the SHR group; ^&^*P* < 0.05, ^&&^*P* < 0.01, and ^&&&^*P* < 0.001 versus the SHR+vehicle group; significantly different as indicated.

Expression of TLR4 protein in the SHR+TLR4-shRNA group was significantly lower than in the SHR and SHR+vehicle groups (Figures 5(a) and 5(b)). There was a significant increase in expression of TLR4, NLRP3, cleaved-caspase-1-p20, TGF-*β*, IL-1*β*, and IL-18 proteins in SHR and SHR+vehicle groups compared with the control group. No differences in protein expression were noted in the SHR and SHR+vehicle groups, while expression of NLRP3, cleaved-caspase-1-p20, TGF-*β*, IL-1*β*, and IL-18 in the SHR+TLR4-shRNA group was clearly reduced compared with the SHR and SHR+vehicle groups (Figures 5(a) and 5(c)–5(h)).

### 3.5. Silencing of TLR4 Inhibited Proliferation of CFs

**Figure 6:**
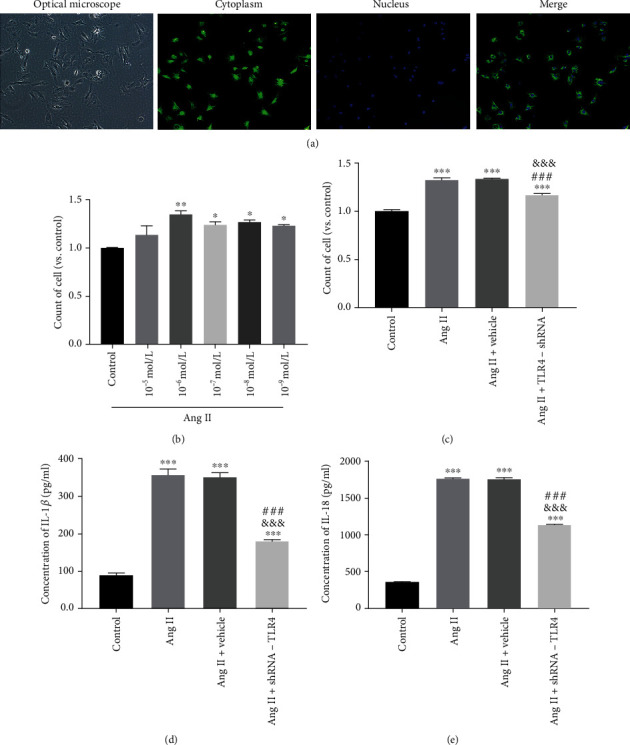
Silencing of TLR4 inhibited proliferation of cardiac fibroblasts (CFs). (a) CF morphology was observed by transmission light microscopy (100x) and immunofluorescence was used to identify vimentin expressed in CFs (100x). (b) CFs were treated with different concentrations of Ang II, and cell proliferation was evaluated using a CCK-8 assay kit (*n* = 3). (c) The effect of TLR4-shRNA on proliferation of cardiac fibroblasts measured by CCK-8 (*n* = 3). (d) The concentration of IL-1*β* in cell culture supernatants was detected by enzyme-linked immunosorbent assay (ELISA) (*n* = 6). (e) The concentration of IL-18 in cell culture supernatants was detected by ELISA (*n* = 6). Data are expressed as mean ± SEM. Statistical analyses: *P* values were evaluated by ANOVA, followed by a post hoc Tukey's test. ^∗^*P* < 0.05, ^∗∗^*P* < 0.01, and ^∗∗∗^*P* < 0.001 versus the control; ^#^*P* < 0.05, ^##^*P* < 0.01, and ^###^*P* < 0.001 versus the SHR group; ^&^*P* < 0.05, ^&&^*P* < 0.01, and ^&&&^*P* < 0.001 versus the SHR+vehicle group; significantly different as indicated.

Under a transmitted light microscope, CFs were densely arranged and typically triangular or star-shaped. Positive expression of vimentin was detected by immunofluorescence, indicating successful isolation of CFs (Figure 6(a)).

It has been reported that Ang II can stimulate cells to proliferate and that the effect depends on Ang II concentrations [26, 27]. Results of the CCK-8 assay showed that cell proliferation tended to increase following 24 h of exposure to Ang II, with the increase being most significant at a concentration of 10^−6^ mol/L (Figure 6(b)). We therefore used 10^−6^ mol/L Ang II to treat cells for 24 h to establish a vitro model of myocardial fibrosis for follow-up experiments. In addition, 10^−6^ mol/L Ang II increased proliferation of CFs in Ang II and Ang II+vehicle groups compared with the control group. CF proliferation was inhibited in the Ang II+TLR4-shRNA group compared with the Ang II and Ang II+vehicle groups (Figure 6(c)).

### 3.6. Silencing of TLR4 Inhibited Activation of NLRP3 Inflammasome and Expression of TGF-*β* In Vitro

Concentrations of IL-1*β* and IL-18 in cell culture supernatants from the Ang II and Ang II+vehicle groups were elevated compared with the control group, indicating successful activation of NLRP3 inflammasome induced by Ang II. Concentrations of IL-1*β* and IL-18 in the Ang II+TLR4-shRNA group were lower than in the Ang II and Ang II+vehicle groups (Figures 6(d) and 6(e)).

**Figure 7:**
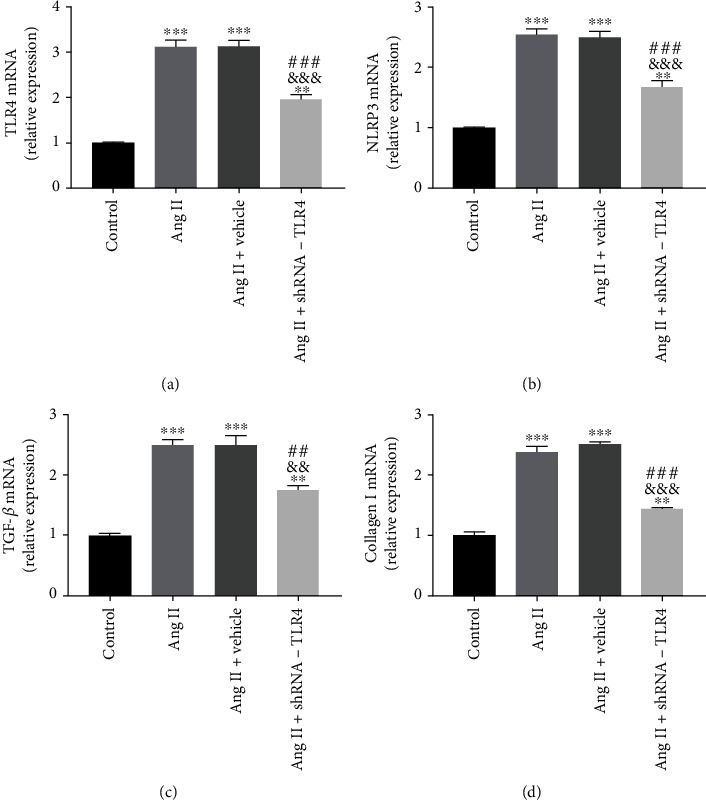
Silencing of TLR4 inhibited mRNA expression of NLRP3 and TGF-*β* in vitro. Relative mRNA expression of TLR4, NLRP3, TGF-*β*, and collagen I normalized to GAPDH in CFs (a–d). All the aforementioned data are represented as mean ± SEM (*n* = 3). Statistical analyses: *P* values were evaluated by ANOVA, followed by a post hoc Tukey's test. ^∗^*P* < 0.05, ^∗∗^*P* < 0.01, and ^∗∗∗^*P* < 0.001 versus the control; ^#^*P* < 0.05, ^##^*P* < 0.01, and ^###^*P* < 0.001 versus the Ang II group; ^&^*P* < 0.05, ^&&^*P* < 0.01, and ^&&&^*P* < 0.001 versus the Ang II+vehicle group; significantly different as indicated.

Expression of TLR4 mRNA in the Ang II+TLR4-shRNA group was significantly lower than in the Ang II and Ang II+vehicle groups, indicating successful lentiviral transfection and knockdown of TLR4 gene expression in the CFs (Figure 7(a)). Compared with the control group, expression of TLR4, NLRP3, TGF-*β*, and collagen I mRNA in the Ang II and Ang II+vehicle groups was significantly higher. Compared with the Ang II and Ang II+vehicle groups, expression of TLR4, NLRP3, TGF-*β*, and collagen I mRNA in the Ang II+TLR4-shRNA group was significantly reduced (Figures 7(b)–7(d)).

**Figure 8:**
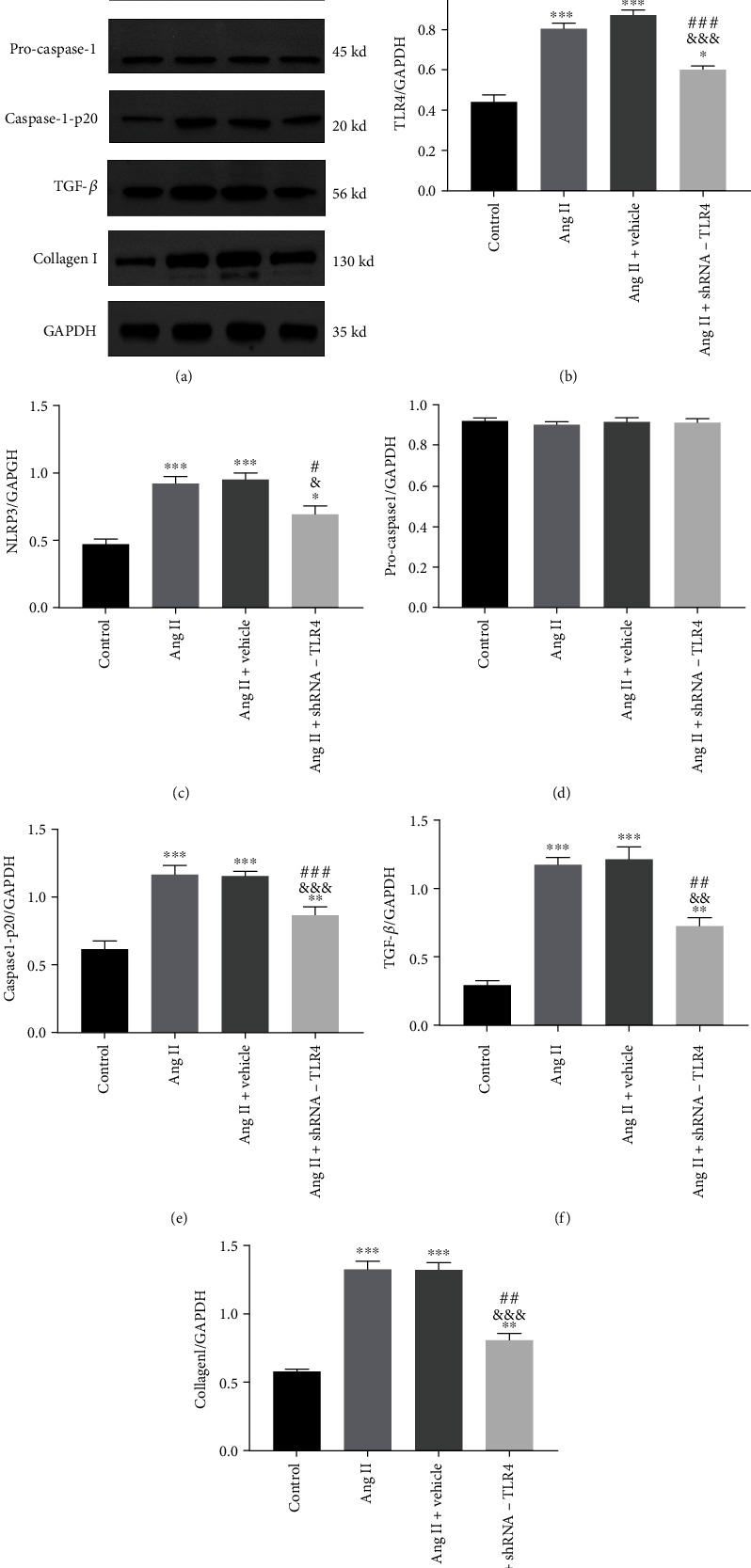
Silencing of TLR4 inhibited expression of NLRP3 and TGF-*β* proteins in vitro. Protein expression of TLR4, NLRP3, caspase-1, TGF-*β*, and collagen I detected by western blots in CFs. (a) Representative western blots show expression of TLR4, NLRP3, pro-caspase-1, caspase-1-p20, TGF-*β*, and collagen I. (b–g) Relative protein expression of TLR4, NLRP3, pro-caspase-1, caspase-1-p20, TGF-*β*, and collagen I normalized to GAPDH in CFs. All the aforementioned data are represented as mean ± SEM (*n* = 3). Statistical analyses: *P* values were evaluated by ANOVA, followed by a post hoc Tukey's test. ^∗^*P* < 0.05, ^∗∗^*P* < 0.01, and ^∗∗∗^*P* < 0.001 versus the control; ^#^*P* < 0.05, ^##^*P* < 0.01, and ^###^*P* < 0.001 versus the Ang II group; ^&^*P* < 0.05, ^&&^*P* < 0.01, and ^&&&^*P* < 0.001 versus the Ang II+vehicle group; significantly different as indicated.

Western blotting showed that levels of TLR4, NLRP3, cleaved-caspase-1-p20, TGF-*β*, and collagen I proteins in the Ang II and Ang II+vehicle groups were clearly higher than in the control group. In addition, expression of TLR4, NLRP3, cleaved-caspase-1-p20, TGF-*β*, and collagen I proteins in the Ang II+TLR4-shRNA group was significantly reduced relative to that in the Ang II and Ang II+vehicle groups (Figure 8).

## 4. Discussion

**Figure 9:**
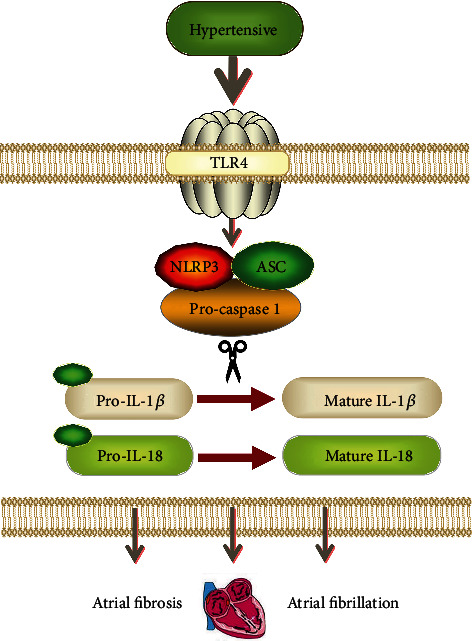
Diagram of the mechanisms investigated in the present study.

This study probed the effect of TLR4 on atrial fibrosis and susceptibility to AF in SHRs and explored a possible mechanism, showing that downregulation of TLR4 inhibits activation of NLRP3 inflammasome, resulting in decreased levels of IL-1*β*, IL-18, and TGF-*β*, and alleviating atrial fibrosis and susceptibility to AF in SHR (Figure 9). Furthermore, we showed that silencing TLR4 in vitro could inhibit the NLRP3 inflammasome and proliferation of CFs.

Matsuda et al. [6] demonstrated the possible involvement of TLR4 in increased oxidative stress, cardiomyocyte hypertrophy, and cardiac dysfunction in patients with Ang II-induced hypertension. Functional TLR4 deficiency has been shown to significantly attenuate cardiac hypertrophy [8, 28]. Consistent with previous research, we further found that silencing TLR4 in the left atrial of SHR alleviated atrial fibrosis and reduced susceptibility to AF. The hypertensive response is preserved or even enhanced in Ang II-infused TLR4-deficient mice [8], consistent with the present results. Some studies have shown that blood pressure is reduced when a TLR4 inhibitor is used [9, 29, 30]. The TLR4 inhibitor could directly inhibit the function of TLR4 channel, thereby lowering blood pressure, while silencing or reducing expression of TLR4 may not affect blood pressure.

Fibroblasts play a critical role in myocardial fibrosis, which is related to cardiac dysfunction [31]. We therefore performed in vitro experiments to investigate the effect of TLR4 on proliferation and collagen synthesis of CFs. Ang II is known to be an important profibrosis factor and is related to myocardial inflammation, both of which are important in myocardial remodeling and heart failure [32, 33]. Ang II triggers biphasic STAT3 activation through TLR4 to initiate cardiac remodeling [34]. We confirmed that Ang II was able to induce proliferation of CFs, and silencing TLR4 could inhibit proliferation and collagen synthesis in CFs induced by Ang II.

The NLRP3 inflammasome, an important component of the innate immune system, is shown to have a mechanistic relationship with AF pathogenesis, and its inhibition may be a potential new approach for treatment of AF [35, 36]. The NLRP3 inflammasome is located downstream of TLR4. It has been reported that suppressing TLR4 can lead to inhibition of the NLRP3 inflammasome-mediated inflammatory response [37]. As reported in previous studies, TGF-*β* has been implicated as a principal mediator of fibrosis associated with inflammation and tissue injury and can be regulated by NLRP3 in fibrotic disease [38, 39]. In our study, silencing TLR4 inhibited activation of the NLRP3 inflammasome and decreased expression of TGF-*β*, leading to reduction of atrial fibrosis and susceptibility to AF in vivo and CF proliferation in vitro. These findings provide novel insights into TLR4's mechanism of action in regulating atrial fibrosis and susceptibility to AF induced by hypertension.

This study had certain limitations. The numbers of rats in each group were relatively small. Moreover, no echocardiogram examination was performed to evaluate changes in atrial structure and function. In addition to the NLRP3 inflammasome, other inflammatory processes, such as NF-*κ*B, STAT3, and MAPK signaling, could be examined further.

## 5. Conclusion

In summary, silencing of TLR4 resulted in inhibition of activation of NLRP3 inflammasome and decreased expression of TGF-*β*, leading to reduction of atrial fibrosis and susceptibility to AF. In vitro, silencing of TLR4 inhibited the NLRP3/TGF-*β* signaling pathway and suppressed CF proliferation and collagen synthesis. We have provided novel insights into the mechanism of atrial fibrosis and AF secondary to hypertension.

## Figures and Tables

**Table 1:** Sequences of q-PCR primers.

Gene	Forward primer	Reverse primer
GAPDH	CATGGTCTACATGTTCCAGT	GGCTAAGCAGTTGGTGGTGC
TLR4	CCGCTCTGGCATCATCTTCA	CCCACTCGAGGTAGGTGTTTCTG
NLRP3	GTGGAGATCCTAGGTTTCTCTG	CAGGATCTCATTCTCGAC
TGF-*β*	CCCCTACATTTGGAGCCTGG	TTGCGACCCACGTAGTAGAC
Collagen I	TGTTGGTCCTGCTGGCAAGAATG	GTCACCTTGTTCGCCTGTCTCAC

## Data Availability

The data used to support the findings of this study are available from the corresponding author upon request.
